# Avocado Oil Improves Mitochondrial Function and Decreases Oxidative Stress in Brain of Diabetic Rats

**DOI:** 10.1155/2015/485759

**Published:** 2015-06-09

**Authors:** Omar Ortiz-Avila, Mauricio Esquivel-Martínez, Berenice Eridani Olmos-Orizaba, Alfredo Saavedra-Molina, Alain R. Rodriguez-Orozco, Christian Cortés-Rojo

**Affiliations:** ^1^Instituto de Investigaciones Químico-Biológicas, Universidad Michoacana de San Nicolás de Hidalgo, 58030 Morelia, MICH, Mexico; ^2^Facultad de Químico Farmacobiología, Universidad Michoacana de San Nicolás de Hidalgo, 58240 Morelia, MICH, Mexico; ^3^Facultad de Ciencias Médicas y Biológicas “Dr. Ignacio Chávez”, Universidad Michoacana de San Nicolás de Hidalgo, 58020 Morelia, MICH, Mexico

## Abstract

Diabetic encephalopathy is a diabetic complication related to the metabolic alterations featuring diabetes. Diabetes is characterized by increased lipid peroxidation, altered glutathione redox status, exacerbated levels of ROS, and mitochondrial dysfunction. Although the pathophysiology of diabetic encephalopathy remains to be clarified, oxidative stress and mitochondrial dysfunction play a crucial role in the pathogenesis of chronic diabetic complications. Taking this into consideration, the aim of this work was to evaluate the effects of 90-day avocado oil intake in brain mitochondrial function and oxidative status in streptozotocin-induced diabetic rats (STZ rats). Avocado oil improves brain mitochondrial function in diabetic rats preventing impairment of mitochondrial respiration and mitochondrial membrane potential (ΔΨ_*m*_), besides increasing complex III activity. Avocado oil also decreased ROS levels and lipid peroxidation and improved the GSH/GSSG ratio as well. These results demonstrate that avocado oil supplementation prevents brain mitochondrial dysfunction induced by diabetes in association with decreased oxidative stress.

## 1. Introduction

Diabetes is characterized by a constant stage of hyperglycemia leading in the long term to severe damage to several systems [1], including the central nervous system (CNS). Diabetes has been involved in several brain conditions such as cerebral ischemia, macrovascular disease, microangiopathy, cognitive decline, and brain atrophy [2]. However, the mechanisms underlying neuronal damage in CNS, known as diabetic encephalopathy, are still unclear [3].

Mitochondrial dysfunction has been hypothesized to be a key factor in the progression of hyperglycemia-mediated neuronal damage [4, 5]. This is related to the large demand of the cells from the CNS for ATP to allow neurotransmission. For this reason, the maintenance of oxidative phosphorylation capacity is extremely important in the CNS since about 90% of the ATP required for the normal function of neurons is provided by mitochondria [6]. Thus, mitochondrial dysfunction may contribute to the loss of neuronal metabolic control and, consequently, to neurodegeneration [7]. This notion is supported by data demonstrating mitochondrial function decline with aging and in age-related diseases, such as diabetes [8].

Mitochondrial alterations related to diabetic encephalopathy include increased mitochondrial fission, excessive ROS levels [9], augmented levels of both lipid peroxidation and nitrite, and decreased levels of total antioxidant [10]. In addition, it has been suggested that diabetes-induced oxidative stress increases the levels of proinflammatory cytokines, which enhances neuronal degeneration [3]. Therefore, mitochondrial oxidative damage contributes, at least in part, to the development of diabetic encephalopathy [11].

Further studies are required about these issues for the development of therapeutic strategies to ameliorate the impact of diabetic encephalopathy and other complications of diabetes. In this regard, nutraceuticals with antioxidant properties have been used as alternative treatments to slow and/or prevent the inherent complications of diabetes [12–14]. A candidate belonging to this group of nutraceuticals is avocado, as this fruit contains a wide variety of antioxidants including carotenoids, tocopherols, chlorophylls, vitamins, and oleic acid (C18:1) as the main fatty acid [15]. Moreover, improvement in glycemic control, plasma lipid profile, and atherogenic index has been observed in diabetic patients consuming avocado in their diets [16]. Regarding the alterations in mitochondrial function and oxidative stress in diabetes, we have previously reported that avocado oil prevented renal mitochondrial dysfunction in streptozotocin-induced type I diabetic rats by preserving the activity of the complex III of the electron transport chain (ETC) and attenuating ROS levels due to protection of the integrity of cytochromes *c* + *c*_1_ [17].

In this work, we aimed to evaluate the effects of avocado oil on brain mitochondrial function and oxidative status in STZ-induced type I diabetic rats. For this purpose, several parameters of mitochondrial function were analyzed such as respiratory control ratio (RCR), activity of the ETC complexes, transmembrane potential (ΔΨ_*m*_), lipid peroxidation levels, ROS levels, and (GSH/GSSG) ratios.

## 2. Materials and Methods

### 2.1. Animals and Experimental Design

Male Wistar rats weighing between 300 and 350 g were used and kept under controlled temperature and 12 hours cycles of light/dark. Rats were feed with a rodent diet and water ad libitum. For the management of the animals, we followed the recommendations from Mexican Federal Regulations for the Use and Care of Animals (NOM-062-ZOO-1999, Ministry of Agriculture, Mexico). This research was also approved by the Institutional Committee for Use of Animals of the Universidad Michoacana de San Nicolás de Hidalgo. Diabetes was induced by intraperitoneal administration of STZ (45 mg/kg), in rats subjected to 12-hour fasting conditions. Five days after diabetes induction, the glucose levels were determined and rats exhibiting blood glucose levels higher than 300 mg/dL were included in the experimental trial.

Rats were randomly divided in four groups: (1) normoglycemic rats (Control); (2) normoglycemic rats plus avocado oil (Control + AO); (3) diabetic rats (Diabetic); (4) diabetic rats plus avocado oil (Diabetic + AO). Avocado oil was administered orally at a dose of 1 mL/250 g weight in a daily basis for a period of 90 days. A commercial presentation of avocado oil (Ahuacatlan, DIRICOM, S.A. de C.V., México), purchased from a local grocery, was used in the experimental trial.

### 2.2. Isolation of Mitochondria

At the end of the treatment, animals were fasted overnight and sacrificed by decapitation. Brain was quickly removed and mitochondria were isolated by differential centrifugation in a Percoll gradient as previously described [18]. Briefly, the entire brain, without the cerebellum, was extracted and placed in a cold medium containing 210 mM mannitol, 70 mM sucrose, 1 mM EGTA, 0.5% bovine serum albumin, and 10 mM MOPS (pH 7.4). The brain was homogenized manually in a glass homogenizer and centrifuged at 400 g. The supernatant was centrifuged at 9000 g. Centrifugations were carried out during 10 min at 4°C. Mitochondrial protein concentration was measured by a modification of the Biuret method [19] calibrated with bovine serum albumin.

### 2.3. Oxygen Consumption

To measure mitochondrial respiration, freshly isolated brain mitochondria were resuspended in a final volume of 2 mL of buffer for measuring oxygen consumption (10 mM HEPES, 100 mM KCl, 3 mM MgCl_2_, and 3 mM KH_2_PO_4_ at pH 7.4) in a sealed glass chamber with constant stirring. The rate of oxygen consumption was determined at room temperature using a Clark-type oxygen electrode coupled to an oxygen monitor YSI 5300 and a computer for data acquisition. The determinations started immediately after adding 10 mM glutamate/malate as respiratory substrate for complex I (state 4) and after 3 minutes, 1 mM ADP was added to determine oxygen consumption in the phosphorylating state (state 3). Finally, inhibitors of complex III (1 *µ*g antimycin A) and complex IV (1 mM KCN) were added to inhibit mitochondrial respiration. The respiratory control ratio (RCR) was calculated from the ratio of the state 3/state 4 respiratory rates.

### 2.4. Determination of Mitochondrial Membrane Potential (ΔΨ_*m*_)

ΔΨ_*m*_ was estimated by a spectrofluorometric assay using Safranin O [20]. 0.5 mg/mL mitochondria were resuspended in a medium containing of 100 mM KCl, 75 mM mannitol, 25 mM sucrose, and 0.05 mM EDTA (pH 7.4). ΔΨ_*m*_ traces were started by measuring basal Safranin O fluorescence during 1 min. Later, mitochondria were energized with 10 mM glutamate/malate and fluorescence changes were followed by additional 4 min. Finally, 5 *µ*M of the uncoupler CCCP (carbonylcyanide-chlorophenylhydrazone) was added to dissipate ΔΨ_*m*_. The changes in Safranin O fluorescence were measured at *λ*_ex_ 495 nm and *λ*_em_ 586 nm in a Shimadzu RF5301PC spectrofluorometer.

### 2.5. Evaluation of Lipid Peroxidation Levels

This determination was carried out in brain mitochondria by measuring the levels of thiobarbituric acid reactive substances (TBARS) [21]. Absorbance was measured at 532 nm with a Perkin Elmer Lambda 18 UV/VIS spectrophotometer. Data were expressed as nanomoles of TBA reactive species (TBARS)/mg protein.

### 2.6. Measurement of the Activity of the ETC Complexes

To determine the activities of the ETC complexes I, II, III, and IV, intact mitochondria were permeabilized with Triton X-100 as previously described [22]. Enzymatic activities were assayed using 0.1 mg/mL permeabilized mitochondria resuspended in 50 mM KH_2_PO_4_ buffer. NADH-oxidoreductase (complex I) activity was assayed in brain mitochondria incubated with 1 *µ*g antimycin A plus 1 mM KCN. After 5 min, 5 mM K_3_Fe(CN)_6_ was added and absorbance was followed during 1 min at 340 nm in a Shimadzu UV2550 spectrophotometer. Then, NADH was added and its oxidation was measured during 4 min. The rate of NADH oxidation was calculated using a molar extinction coefficient of 16.3 mM^−1^ cm^−1^ for NADH [23]. Succinate-DCIP oxidoreductase (complex II) activity was measured spectrophotometrically at 600 nm by following the reduction of 2,6-dichlorophenolindophenol (DCIP). Antimycin A-sensitive succinate-cytochrome *c* oxidoreductase (complex III) activity was followed by measuring at 550 nm the reduction of cytochrome *c*. Cytochrome *c* oxidase (complex IV) activity was evaluated by measuring the oxidation of reduced cytochrome *c* at 550 nm [24].

### 2.7. Measurement of ROS Levels

ROS levels were determined by measuring the oxidation of 2′,7′-dichlorodihydrofluorescein diacetate (H_2_DCFDA). 0.5 mg/mL intact mitochondria and 1.25 mM H_2_DCFDA were incubated in a buffer containing 10 mM HEPES, 100 mM KCl, 3 mM MgCl_2_, and 3 mM KH_2_PO_4_ (pH 7.4) during 20 min at 4°C under constant shaking. Later, mitochondrial suspension was placed in a quartz cuvette and basal fluorescence was recorded. After 1 min, 10 mM glutamate/malate was added and the changes in H_2_DCFDA fluorescence were further followed by 20 min [17]. Fluorescence changes were detected in a Shimadzu RF-5301PC spectrofluorophotometer (*λ*_ex_ 485 nm; *λ*_em_ 520 nm).

### 2.8. Glutathione Assay

Mitochondrial samples were treated with 5% (v/v) sulfosalicylic acid and centrifuged at 7800 g for 10 min to remove denatured proteins, and reduced glutathione (GSH) and oxidized glutathione (GSSG) were determined by an enzymatic method. The total glutathione (GSH + GSSG) content was assayed in a cuvette containing 90 *μ*L of the supernatant in 0.1 M sodium phosphate buffer (pH 7.5), 3 mM 5,5′-dithiobis(2-nitrobenzoic acid) (DTNB) and 0.115 unit/mL glutathione reductase in a final volume of 1 mL. After 5 min of incubation at room temperature, 2 mM NADPH was added and the kinetics of the reaction was monitored for 5 min. The increment in absorbance at 412 nm was converted to GSH concentration using a standard curve with known amounts of GSH [25]. For determination of GSSG, the same DTNB recycling assay was performed after using 3% (v/v) 4-vinylpyridine to remove reduced GSH followed by incubation at room temperature for 1 h before the beginning of the assay.

### 2.9. Data Analysis

Results are expressed as the mean ± standard error of at least 3 independent experiments using samples from different animals for each experiment. Statistical differences of the data were determined with Student's *t*-test using Sigma Plot software v11.0.

## 3. Results

### 3.1. Body Weight and Levels of Serum Glucose and Lipids

The brains used for the experiments of this study were dissected from the same rats used for other study reporting the effects of avocado oil on liver mitochondrial function of diabetic rats [26]. As reported in that study, control and STZ rats exhibited at the end of the trial fasting serum glucose levels of 47.5 ± 4.1 and 367.6 ± 8.0 mg/dL, respectively. Cholesterol and triglycerides levels reached 70.5 ± 5.0 and 89.8 ± 13.99 mg/dL, respectively, in control animals and 94.2 ± 4.5 and 396.5 ± 33.6 mg/dL, respectively, in STZ rats. Avocado oil normalized cholesterol and decreased triglyceride levels in STZ-treated rats, as the levels of these lipids were 60.9 ± 4.6 and 131.8 ± 21.2 mg/dL, respectively. Avocado oil had not any effect on glucose levels of STZ-treated rats. Moreover, control and STZ-treated rats displayed at the end of the study body weight values of 502.3 ± 9.6 and 246.3 ± 16.0 g, respectively, without avocado oil treatment significantly altering this parameter neither in control nor in STZ-treated rats. Together, these results confirm that STZ treatment induced diabetes and demonstrate that avocado oil corrects only diabetic dyslipidemia.

### 3.2. Effects of Diabetes and Avocado Oil on Respiratory Function of Brain Mitochondria

**Figure 1:**
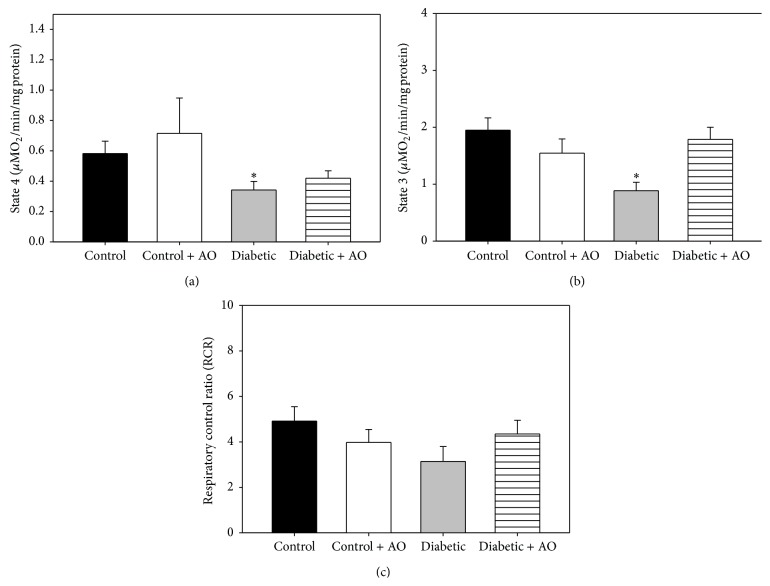
Effects of avocado oil treatment in brain mitochondrial respiratory chain parameters: (a) state 3 respiration; (b) state 4 respiration, and (c) respiratory control ratio (RCR). Data are the mean ± EE of *n* = 5. ^*∗*^ *p* < 0.05 compared with brain control mitochondria.

Oxygen consumption rates (OCR) in resting state (state 4) and phosphorylating state (state 3) were measured with the objective to evaluate whether diabetes impaired mitochondrial function and determine the protective effects of avocado oil. Diabetes had a notable impact on brain mitochondria respiration (Figures 1(a) and 1(b)) as OCR in states 4 and 3 decreased 41.3% and 54.5%, respectively. Importantly, avocado oil prevented these effects, being this protective effect more prominent in state 3 respiration (Figure 1(b)). Impaired respiratory rates led to lower RCR in diabetic rats (Figure 1(c)), although, in this case, RCR values were not different in a statistically significant way.

### 3.3. Effects of Diabetes and Avocado Oil on Mitochondrial Transmembrane Potential (ΔΨ_*m*_)

**Figure 2:**
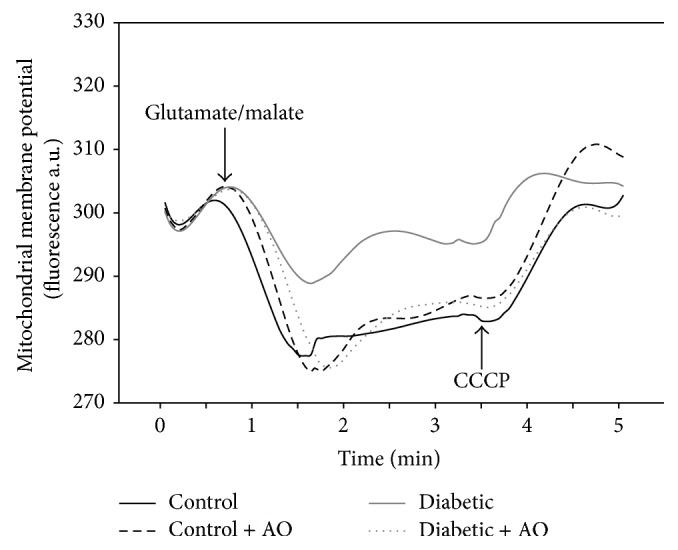
Effect of avocado oil on mitochondrial membrane potential (ΔΨ_*m*_). Representative traces of fresh isolated brain mitochondria from diabetic rat. Membrane potential was expressed in fluorescence arbitrary units (a.u.). The traces are typical of four experiments.

Further characterization of impaired brain mitochondrial function in diabetic rats was carried out by analyzing ΔΨ_*m*_. As observed in Figure 2, the energization of mitochondria from the control group (black line) with glutamate/malate elicited a large, instantaneous decrease in Safranin O fluorescence, which reflects the establishment of the ΔΨ_*m*_. Moreover, ΔΨ_*m*_ remained stable after 2 min and the addition of an uncoupler (CCCP) induced an increase in Safranin fluorescence at initial levels before substrate addition, which is indicative of full dissipation of the ΔΨ_*m*_. In contrast, the changes elicited by glutamate/malate in Safranin fluorescence were of a considerably lower magnitude in mitochondria from diabetic rats (gray line) and occurs at a slower rate than in mitochondria from control rats (black line), which together indicates that diabetes impaired brain mitochondrial functionality. Notably, avocado oil intake fully prevented the alterations in the ΔΨ_*m*_ observed in diabetic rats (gray pointed line) and did not alter this parameter in the control group (black discontinuous line). Therefore, these results confirm that diabetes induced brain mitochondrial dysfunction and that avocado oil fully prevented this alteration.

### 3.4. Analysis of the Effects of Diabetes and Avocado Oil on ETC Functionality

**Figure 3:**
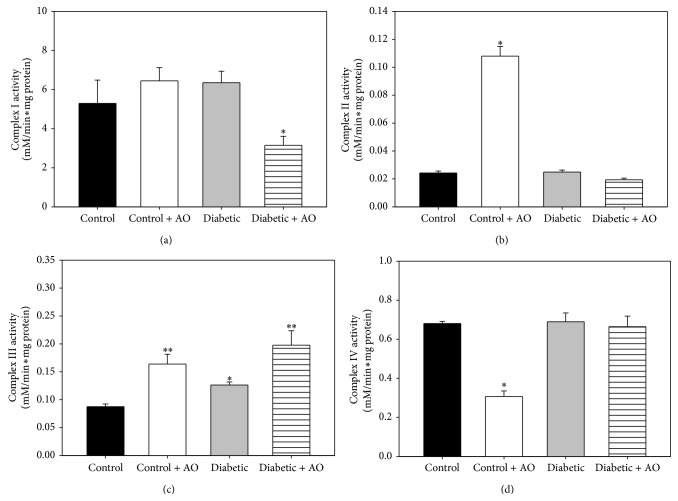
Effect of avocado oil in complexes I (a); II (b); III; (c) and IV (d) activities in brain mitochondria. Data are the mean ± EE of (*n* = 4–8). ^*∗*^ *p* < 0.05, ^*∗∗*^ *p* < 0.01 compared with brain control mitochondria.

The activities of the complexes from the ETC are shown in Figure 3. Regarding complex I, no differences in activity (Figure 3(a)) were observed between the control and diabetic groups; however, avocado oil intake decreased 40.5% of this activity in comparison with the control group. Diabetes did not provoked changes in complex II activity (Figure 3(b)), but avocado oil intake in control rats induced an increase of 3.2-fold in this activity. Regarding the activity of the complex III (Figure 3(c)), diabetes enhanced this activity by 44.2% when compared to control group. Avocado oil also augmented the activity of complex III in mitochondria from diabetic and normoglycemic rats in 125.7% and 87.1% respectively. Complex IV activities were similar in the mitochondrial from all groups, except by the control group treated with avocado oil, which exhibited a decrease of 54.9% in complex IV activity.

### 3.5. Influence of Diabetes and Avocado Oil on ROS Levels

**Figure 4:**
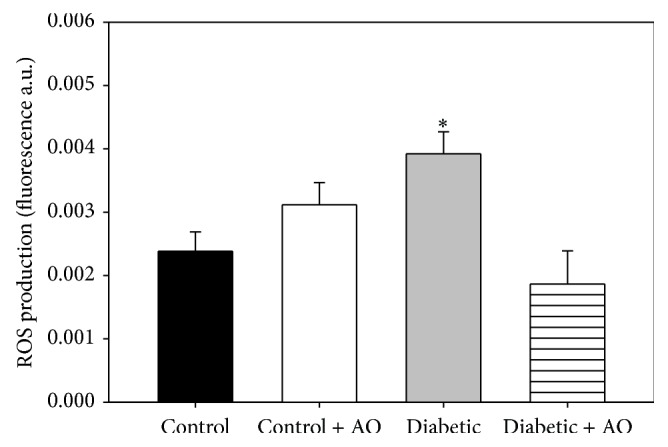
Effect of avocado oil in brain mitochondria ROS levels. Experiment was carried out using 10 mM glutamate/malate as a mitochondria ETC substrate. ROS levels were expressed in fluorescence arbitrary units (a.u.). Data are the mean ± EE of (*n* = 5-6). ^*∗*^ *p* < 0.05 compared with brain control mitochondria.

In order to explore whether the impairment in both ΔΨ_*m*_ and respiration was related to enhanced ROS levels, we evaluated the changes in the fluorescence of H_2_DCFDA in response to the addition of glutamate/malate. The data presented in Figure 4 shows that ROS levels were increased by 64.5% in brain mitochondria from diabetic rats when compared to control rats. It was also observed that avocado oil administration fully prevented this effect in mitochondria from diabetic rats, while in mitochondria from control rats, avocado oil did not altered ROS levels.

### 3.6. Effects of Diabetes and Avocado Oil on Brain Mitochondria Oxidative Stress

**Figure 5:**
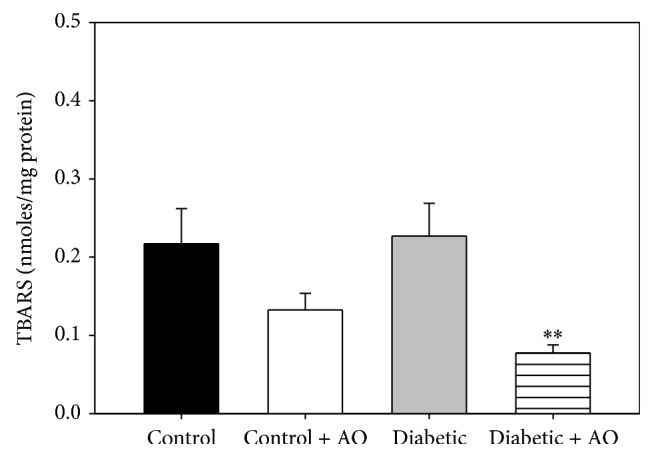
Effect of avocado oil on TBARS of brain mitochondria. Data are the mean ± EE of (*n* = 4). ^*∗∗*^ *p* < 0.01 compared with brain control mitochondria.

Lipid peroxidation and GSH/GSSG ratios were analyzed as markers of oxidative stress to test whether enhanced ROS levels in diabetic rats and the protection conferred by avocado oil were parallel to changes in oxidative stress. In comparison to control rats, the levels of TBARS were similar in mitochondria from diabetic rats (Figure 5). However, avocado oil decreased the levels of lipid peroxidation in both control and diabetic groups, although this effect was statistically significant only in the diabetic group (65%).

Regarding to the redox status of glutathione, the GSH/GSSG ratio of mitochondria from diabetic rats was 38.3% lower with respect to mitochondria from control animals, indicating a state of higher oxidative stress in mitochondria from diabetic animals. Avocado oil prevents this effect and even augmented by 3.1-fold the GSH/GSSG ratio in comparison to mitochondria from control animals. Besides, avocado oil also produced a more discrete, 1.3-fold increase in this parameter in normoglycemic rats.

## 4. Discussion

Diabetes complications are associated with end-stage damage in the eyes, kidneys, peripheral nerves, and the brain [27–29]. In the CNS, type 1 diabetes encephalopathy is manifested like cognitive dysfunction characterized by a slowing of mental speed and a diminished mental flexibility [30]. Furthermore, the risk of dementia appears to be almost doubled in diabetic patients [31]. The exact mechanisms underlying the complications in CNS occurring in diabetes are not fully understood [32], as it seems to be a complex, multifactorial process including physiological, molecular, and metabolic alterations [28]. Due to the fact that avocado oil administration decreases oxidative stress and protects mitochondrial function in kidney mitochondria [16], it was decided to evaluate its effect on the CNS, with a focus on bioenergetics and oxidative stress of brain mitochondria from type I diabetic STZ-induced rats.

Mitochondrial dysfunction has been proposed to mediate development of diabetes complications in many tissues including neurons [33]. Our results show that diabetes impairs brain mitochondrial respiration in both state 4 and state 3 (Figure 1), which may be interpreted as diabetes induced alterations in the functioning of the ETC and/or in oxidative phosphorylation like in other tissues such as cardiac muscle, liver, and kidney [34–36]. This is consistent with reports demonstrating mitochondrial dysfunction in the brain of diabetes models [37, 38]. A probable factor leading to decreased respiration might be declined expression of nuclear respiratory factor 1 (NRF-1) and peroxisome proliferator-activated receptor-*γ* coactivator 1*α* (PGC-1*α*), which regulates mitochondrial biogenesis and the expression of several components of the ETC [39]. Proteomic studies also have shown a reduction in the expression and activity of some components of the ETC, contributing further to neuronal mitochondrial dysfunction [40]. Another characteristic of diabetes is the increased oxidative stress, which also directly affects mitochondrial function by affecting lipids, membrane proteins, and mitochondrial DNA [41]. Our results of impaired respiration in state 3 imply a decrease in oxidative phosphorylation of ADP in mitochondria of diabetic rats. Oxidative phosphorylation depends importantly on ΔΨ_*m*_ to drive the conversion of ADP into ATP. As ΔΨ_*m*_ was severely impaired in mitochondria of diabetic rats (Figure 2), our results suggest that depolarization of the inner mitochondrial membrane leads to lower rates of respiration in state 3, although the possibility that other factors like impaired activity and/or expression of both the F_1_F_0_-ATP synthase and the adenine nucleotide translocator may be also involved in this phenomena remains.

**Figure 6:**
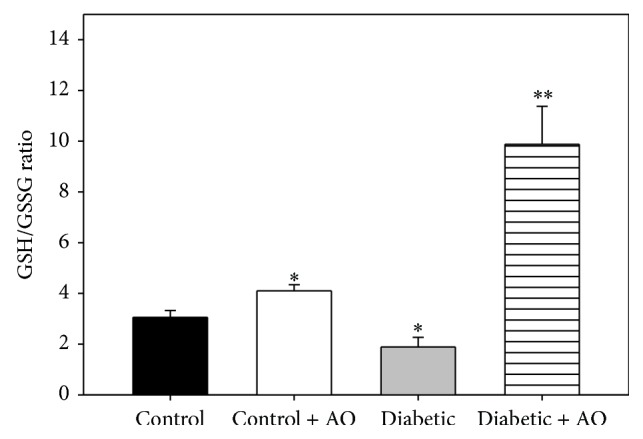
Effect of avocado oil on GSH/GSSG ratio in brain mitochondria. Data are the mean ± EE of (*n* = 4). ^*∗*^ *p* < 0.05, ^*∗∗*^ *p* < 0.01 compared with brain control mitochondria.

The protective effects of avocado oil on mitochondrial respiration and ΔΨ_*m*_ during diabetes may be attributed to some of the compounds present in the oil, such as lutein [15] as this carotenoid interacts with transcription factors counteracting mitochondrial dysfunction such as NRF-1 and PGC-1*α* [42, 43]. Another probable mechanism involved may be decreased mitochondrial oxidative stress, as avocado oil had an antioxidant effect in mitochondria by reducing the levels of lipid peroxidation (Figure 5) ROS levels (Figure 4) and by maintaining the mitochondrial redox state to similar levels that control brain mitochondria (Figure 6), which may further contribute to preserve adequate mitochondrial function.

Regarding the activities of the complexes from the ETC, the only significant change during diabetes was an increase in the activity of complex III (Figure 3), which has been reported for some brain regions such as the prefrontal cortex and striatum of diabetic rats [5]. This increase in activity may be related to a compensatory effect in an effort to counteract enhanced ROS levels and decreased expression of the proteins constituting the ETC [15], including some subunits of complex III [44] and diminished amounts of cytochrome *b* and *c* + *c*_1_ in brain mitochondria-STZ rats [45]. Avocado oil further increased the activity of the complex III in the diabetic rats, which may contribute to improved electron flow through the redox centers of complex III, as evidenced by improved respiration in diabetic mitochondria treated with avocado oil. Enhanced complex III activity may reduce the half-life of semiquinone intermediates and decrease ROS levels as semiquinones are electron donors to oxygen to form superoxide anion (O_2_^∙−^). This hypothesis is in fully agreement with ameliorated ROS levels (Figure 4) and decreased oxidative stress (Figure 6) observed in mitochondria from diabetic rats treated with avocado oil. On the other hand, decreased activity of complex I in diabetic rats supplemented with avocado oil might contribute to decreased ROS levels due to electron leak at the ETC by limiting the oxidation of NADH and decreasing the rate of semiquinone generation, which in turn agrees with the role of the complex I also in ROS generation besides complex III [46, 47]. On the other hand, it would be argued that the lack of inhibitory effects of diabetes on the activities of the ETC complexes does not fit well with decreased respiratory rates and partially dissipated ΔΨ_*m*_ observed in mitochondria of the same animals. However, as pointed out by Brand and Nicholls, [48], mitochondrial function (i.e., substrate oxidation and ATP turnover) is not only under the control of the ETC enzymes but is widely shared between many processes, including adenine nucleotide exchange by adenine nucleotide translocase, phosphate availability due to phosphate carrier activity or F_1_F_0_-ATP synthase activity. For this reason, altered ETC functionality may indeed have a negligible effect on overall mitochondrial function. Based on these considerations, it may be suggested that mitochondrial dysfunction in brain mitochondria is independent of alterations on the activities of the ETC enzymes. Instead, decreased respiration in state 3 may be due to alterations in ADP phosphorylation as was discussed above.

Mitochondrial ROS levels were higher in the diabetic group (Figure 4). In other studies, increased ROS levels have been described to activate signaling pathways that lead to cell death of neurons and the development of diabetic encephalopathy [4, 49]. Moreover, avocado oil consumption prevented exacerbated ROS generation in diabetic rats, which raises the possibility that the onset of diabetic encephalopathy might be delayed in these animals. The observation that avocado oil decreases mitochondrial ROS levels confirms its antioxidant potential. Its antioxidant capacity may be also related to its constitution of fatty acids since monounsaturated fatty acids, as oleic acid (C18:1), which comprises ~60% of the fatty acids present in the oil, are less susceptible to damage by ROS that polyunsaturated fatty acids [50]. It is also possible that many of the antioxidant compounds in avocado such as vitamins, carotenoids, chlorophylls, and tocopherols are present in the oil and all these components could be responsible for the observed effect, where the lipophilic components allow accumulation in mitochondria. This feature has been used for the synthesis of new mitochondria-target antioxidants [51], for example, the SOD-mimics [52], which have been created to reduce ROS in oxidative stress related diseases like diabetes.

To estimate the extent of mitochondrial oxidative stress during diabetes and the ability of avocado oil to counteract this process, lipid peroxidation also was determined. According to different reports about the levels of lipid peroxidation in brain mitochondria, there is no consensus about whether this process remains unaltered or increases during diabetes [37, 41, 53]. A probable answer to this issue is that increased lipid peroxidation occurs differentially only in certain brain regions such as the prefrontal cortex and amygdala [10, 38]. In our experiments, an increase in TBARS levels in mitochondria of diabetic group was not observed (Figure 5), which might be related with the fact that we used a whole brain homogenate to isolate mitochondria. Avocado oil decreased lipid peroxidation of mitochondrial membranes of the brain from control and diabetic rats. This probably reflects an antioxidant effect in the entire brain. Diabetes also decreased the GSH/GSSG ratio, which, in contrast with the above controversy about lipid peroxidation, is consistent with other reports where, besides, the total glutathione content was found to be decreased in diabetic brain [3, 38]. Lower GSH/GSSG ratios impair the ability of some antioxidant enzymes that need GSH to regenerate its function, which further increases oxidative stress in brain diabetic mitochondria.

It is important to note that one important limitation of this work is that cognitive decline due to diabetes and the impact of avocado oil were not assessed in this study, which impedes to confer avocado oil a protective role on diabetic encephalopathy. As a first approach, it was intended in this study to explore whether avocado oil supplementation may protect from diabetes-induced brain mitochondrial dysfunction and exacerbated oxidative stress. However, we thought that the findings from this study pave the road to further research addressing if the administration of avocado oil may have beneficial effects in diabetic encephalopathy, since the attenuation of some features of oxidative stress observed in our study, such as increased lipid peroxidation and glutathione exhaustion, is believed to ameliorate cognitive deficits during diabetes [54].

## 5. Conclusion

Avocado oil improves brain mitochondrial function in diabetic rats by preventing the impairment in mitochondrial respiration and ΔΨ_*m*_ induced by diabetes, besides increasing complex III activity. This may be related to decreased ROS levels and improved redox status in diabetic rats as reflected by a higher GSH/GSSG ratio. These effects might delay the onset of diabetic encephalopathy, but this possibility remains to be investigated.
